# Pancytopenia and Methemoglobinemia in a Patient With Multiple Myeloma Presenting With Syncope and Dyspnoea: A Diagnostic Challenge

**DOI:** 10.7759/cureus.106950

**Published:** 2026-04-13

**Authors:** Akram Abdallah, Rawan Honeini, Pemba Tamang, Mahmoud Abughazal, Dia Mobaideen

**Affiliations:** 1 Emergency Medicine, Pilgrim Hospital, United Lincolnshire Teaching Hospitals National Health Service Trust (ULHT), Boston, GBR; 2 Emergency Medicine, United Lincolnshire Teaching Hospitals National Health Service Trust (ULHT), Boston, GBR; 3 Acute Medicine, United Lincolnshire Teaching Hospitals National Health Service Trust (ULHT), Boston, GBR; 4 Acute Medicine, Lincoln County Hospital, Lincoln, GBR

**Keywords:** chronic anaemia, diagnosis of multiple myeloma, hypoxia, laboratory findings of pancytopenia, methemoglobinemia

## Abstract

Methemoglobinaemia is a rare but important cause of hypoxia that may be overlooked, particularly in patients with complex comorbidities. We present a 76-year-old man with Multiple Myeloma undergoing chemotherapy who presented with recurrent syncope and exertional dyspnoea. Initial investigations revealed pancytopenia and hypoxia. Imaging excluded pulmonary embolism and infection. Arterial blood gas analysis demonstrated elevated methemoglobin levels (10.5%), confirming methemoglobinaemia. The patient was managed with supportive therapy, including oxygen, blood transfusion, and filgrastim, resulting in clinical improvement. This case highlights the importance of considering methemoglobinaemia in patients with unexplained hypoxia, especially when conventional investigations are unremarkable. The coexistence of severe anaemia can further exacerbate tissue hypoxia and complicate diagnosis.

## Introduction

Hypoxia is a common presentation in emergency and acute medical settings and is most frequently attributed to cardiopulmonary causes such as pneumonia or pulmonary embolism. However, less common aetiologies, including methaemoglobinaemia, should be considered when initial investigations are inconclusive. Methaemoglobinaemia results from oxidation of haemoglobin iron from the ferrous (Fe²⁺) to the ferric (Fe³⁺) state, which impairs oxygen binding and delivery and leads to functional anaemia [1,2]. Although it is most commonly drug-induced, associated with agents such as local anaesthetics, nitrates, and certain antimicrobials, cases without an identifiable trigger have been reported, particularly in the context of systemic illness, malignancy, or increased oxidative stress [3,4].

Patients with multiple myeloma frequently develop anaemia due to marrow infiltration, reduced erythropoiesis, and treatment-related cytopenias, all of which can exacerbate tissue hypoxia [5]. In such patients, even modest elevations in methaemoglobin levels may have a disproportionate clinical impact due to reduced overall oxygen-carrying capacity. This case highlights the diagnostic challenge of hypoxia resulting from the coexistence of anaemia and methaemoglobinaemia, emphasising the importance of considering dyshemoglobinaemias when there is a mismatch between oxygen saturation and arterial oxygen tension.

## Case presentation

A 76-year-old man presented with a two-day history of recurrent syncopal episodes accompanied by dizziness and exertional dyspnoea, with symptoms improving at rest but progressively worsening over time. He denied chest pain, cough, or fever. His medical history was significant for multiple myeloma on daratumumab-based chemotherapy (DRD regimen), chronic kidney disease stage 3, hypertension, and previous prostate cancer. The patient's medication regimen consists of allopurinol 300 mg, amlodipine 10 mg, and ezetimibe 10 mg, all taken as single tablets once a day, in addition to apixaban 2.5 mg and acyclovir 200 mg twice a day. On presentation, his vital signs were stable, with a pulse of 70 bpm (beats per minute), respiratory rate of 18 breaths per minute, temperature of 36.3°C, blood pressure of 124/64 mmHg, and oxygen saturation of 92% while receiving 4 L of oxygen.

Laboratory investigations (Table 1) demonstrated pancytopenia, with haemoglobin level 77 g/L, white cell count 2.4 ×10⁹/L, and platelet count of 77 ×10⁹/L. Red cell indices showed macrocytosis (MCV 105 fL), and blood film revealed teardrop poikilocytosis. Inflammatory markers were low, and blood and sputum cultures were negative.

**Table 1:** Lab parameters Hb: hemoglobin; WBC: white blood cell count; Cr: creatinine; Na: sodium; K: potassium; GFR: glomerular filtration rate.

Lab Parameters	Value	Normal Range
WBC	2.4	4.5-11 x 10^9^/L
Platelets	77	150-400 x 10^3^/µL
Urea	4.9	2.1-8.5 mmol/L
Cr	82	59-104 μmol/L
Na	140	136-145 mmol/L
Hb	7.7	14-18 g/dL
K	3.6	3.6-5.2 mmol/L
GFR	80	90-100 ml/min

Arterial blood gas analysis demonstrated a methemoglobin level of 10.5% (Table 2). A repeat venous blood gas performed six hours after initiation of oxygen therapy (Table 3) showed a normal pH with a reduced but still elevated methemoglobin level of 8.8%, consistent with methemoglobinaemia. A key observation was the discrepancy between oxygen saturation and the relatively preserved partial pressure of oxygen, prompting further investigation.

**Table 2:** Initial arterial blood gas analysis of the patient in the accident and emergency department * indicates results are outside the normal range

Parameter	Measured value	Normal range
pH	7.44	7.350-7.450
Carbon dioxide partial pressure (pCO₂)	4.96	4.30-6.40 kPa
Oxygen partial pressure (pO₂)	23	1 11.0-14.4 kPa
Lactate	1.3	1.0-1.8 mmol/L
Total hemoglobin	81*	117-170 g/L
Saturated oxygen (SpO₂)	89*	94.0-98.0%
Oxyhemoglobin	88 *	94.0-98.0%
Carboxyhemoglobin	0	0.0-3.0%
Methemoglobin	10.5 *	0.0-1.5%
Deoxyhemoglobin	2	0.0-3.0%
Actual base excess	0.3	-2.0-2.0 mmol/L
Actual bicarbonate	25.2	22.0-29.0 mmol/L
Temperature	37°C	-
Fraction of inspired oxygen (FiO₂)	21%	-

**Table 3:** Venous blood gas analysis of the patient in the Accident and Emergency Department six hours after oxygen therapy * indicates results are outside the normal range

Parameter	Measured value	Normal range
pH	7.396	7.350-7.450
Carbon dioxide partial pressure (pCO₂)	5.47	4.30-6.40 kPa
Oxygen partial pressure (pO₂)	3.77	11.0-14.4 kPa
Lactate	1.3	1.0-1.8 mmol/L
Total hemoglobin	81*	117-170 g/L
Saturated oxygen (SpO₂)	41.1 *	94.0-98.0%
Oxyhemoglobin	36.9 *	94.0-98.0%
Carboxyhemoglobin	1.3	0.0-3.0%
Methemoglobin	8.8 *	0.0-1.5%
Deoxyhemoglobin	53.0 *	0.0-3.0%
Actual base excess	0.3	-2.0-2.0 mmol/L
Actual bicarbonate	25.2	22.0-29.0 mmol/L
Temperature	37°C	-
Fraction of inspired oxygen (FiO₂)	21%	-

**Figure 1:**
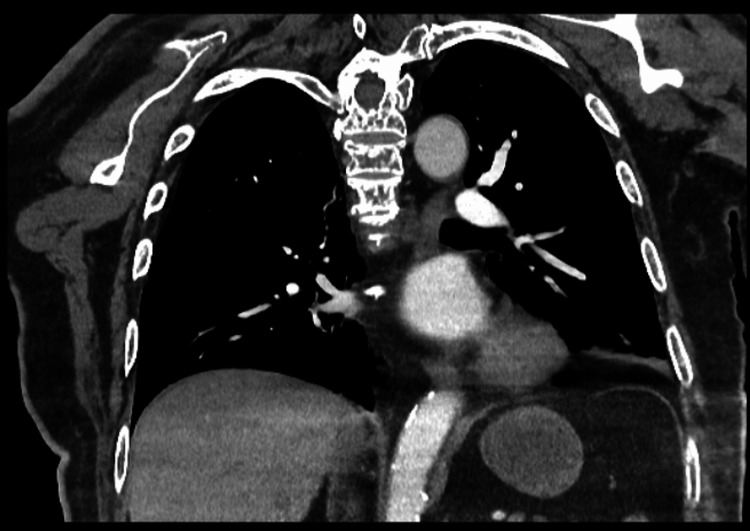
Contrast-enhanced CT of the pulmonary arteries (coronal view) The CT image shows a few thick, scattered linear atelectatic strands in the bilateral lung bases.

**Figure 2:**
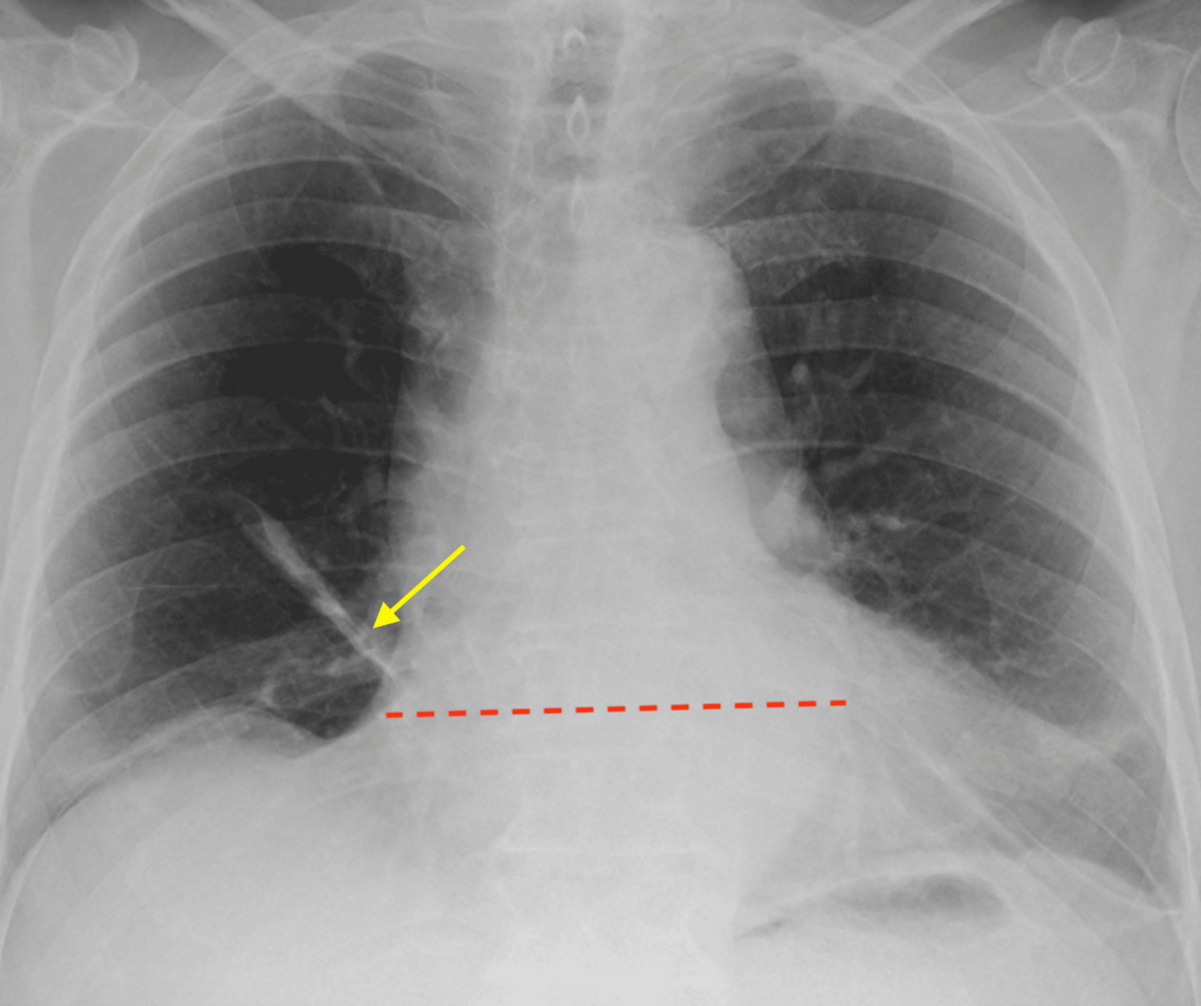
Bibasal linear atelectasis chest X-ray of the patient Yellow arrow denote linear atelectasis; the dotted red line measures the enlarged cardiac silhouette (cardiomegaly).

CT pulmonary angiography (Figure 1) revealed no evidence of pulmonary embolism, while chest X-ray (Figure 2) showed cardiomegaly with bibasal atelectasis and no signs of consolidation or pleural effusion.

The patient was admitted under the medical team for seven days and managed with supportive and targeted therapies, including supplemental oxygen, intravenous empirical antibiotics due to neutropenia, blood transfusion for anaemia, and filgrastim for neutropenia. The initial arterial blood gas (ABG) at the Emergency Department (ED) triage showed a methaemoglobin level of 10.5%. After a few hours of oxygen therapy and reassessment by another doctor, the level decreased to 8.8%. When the ED referred the patient to the medical team, it was decided that methylene blue was not required at that stage. According to guidelines from Toxbase, treatment with methylene blue is generally indicated for patients with methemoglobin levels reaching 20-30% if they are asymptomatic. However, in cases where patients exhibit symptoms, intervention may be necessary at much lower concentrations, specifically within the 10-15% range [3]. Hence, no specific antidotal therapy, such as methylene blue, was required, as methaemoglobin levels were moderate and the patient improved with supportive care alone. He showed clinical improvement with resolution of dizziness and improvement in dyspnoea, with reduced oxygen requirements and stable haemodynamics throughout admission. He was subsequently discharged with continuation of chemotherapy and planned outpatient follow-up.

## Discussion

This case highlights methemoglobinaemia as a rare but important cause of hypoxia, particularly in patients with complex haematological conditions. Methemoglobinaemia is an uncommon but well-recognised cause of impaired oxygen delivery, often presenting a diagnostic challenge due to non-specific symptoms and the presence of more common differentials such as infection, pulmonary embolism, or anaemia, especially when imaging findings are normal [4]. A key diagnostic clue is the discrepancy between oxygen saturation measured by pulse oximetry and arterial oxygen tension, which is characteristic of dyshemoglobinaemias.

Pulmonary embolism (PE) was initially a primary diagnostic consideration given the patient's active malignancy and the use of the DRD regimen, both of which significantly increase the risk of venous thromboembolism. However, this was effectively ruled out after the CT Pulmonary Angiogram (Figure 1) showed no evidence of a clot. In this case, a cardiogenic cause of syncope, such as a significant arrhythmia, is considered unlikely as the patient's ECG demonstrated a normal sinus rhythm with a stable heart rate of 83 bpm. While the presence of cardiomegaly on imaging suggests some underlying structural heart disease, the lack of acute ischemic changes or conduction abnormalities on the ECG points away from a primary cardiac event as the immediate trigger for his symptoms.

Instead, the clinical picture leans more toward a combination of hematologic and dyshemoglobinemic factors. The severe anemia significantly reduced his oxygen-carrying capacity, while the methemoglobinemia (10.5%) further impaired oxygen delivery to tissues. This cumulative "hypoxic hit" likely exceeded his physiological threshold, resulting in the exertional dyspnea and syncopal episodes, particularly when his cardiac output could not compensate for the reduced oxygen content in the blood. No clear pharmacological trigger was identified. Common causes of acquired methemoglobinaemia include oxidising agents such as local anaesthetics, nitrates, and certain antibiotics; however, medications such as allopurinol, acyclovir, and paracetamol are not typically implicated, suggesting a multifactorial aetiology [5].

Chemotherapy-related oxidative stress may have contributed to haemoglobin oxidation in this patient. Individuals with haematological malignancies, including multiple myeloma, often exhibit impaired erythropoiesis and altered red cell integrity, which can increase susceptibility to oxidative damage [6]. Additionally, bone marrow dysfunction may further reduce the capacity to compensate for oxidised haemoglobin. Multiple myeloma is associated with increased oxidative stress and reactive oxygen species production, which can overwhelm erythrocyte antioxidant defenses and promote haemoglobin oxidation to methaemoglobin; red blood cells are particularly susceptible to oxidative injury due to limited repair capacity and reliance on redox systems [7]. However, the DRD regimen (daratumumab, lenalidomide, and dexamethasone) is not typically associated with methemoglobinemia, and there are no well-established reports or guideline warnings linking these therapies to the condition [8].

Severe anaemia, as observed in this case (haemoglobin as low as 77 g/L), likely compounded the physiological impact of methemoglobinaemia. Even relatively low levels of methemoglobin can significantly impair oxygen delivery when total haemoglobin is markedly reduced, creating a “dual-hit” effect [9]. Overall, this case underscores the importance of considering non-pulmonary causes of hypoxia and recognising methemoglobinaemia as a potential contributor, particularly in patients with complex haematological disorders and unexplained oxygen desaturation.

## Conclusions

Methemoglobinemia should be considered in patients with unexplained hypoxia, especially when imaging is unremarkable. Coexisting anaemia can significantly worsen tissue hypoxia. Arterial blood gas with co-oximetry is essential for diagnosis. Early recognition and supportive management can lead to favourable outcomes.
